# Highly Selective and Stable Cu Catalysts Based on Ni–Al Catalytic Systems for Bioethanol Upgrading to n-Butanol

**DOI:** 10.3390/molecules28155683

**Published:** 2023-07-27

**Authors:** Yan Xiao, Nannan Zhan, Jie Li, Yuan Tan, Yunjie Ding

**Affiliations:** 1 Hangzhou Institute of Advanced Studies, Zhejiang Normal University, 1108 Gengwen Road, Hangzhou 311231, China; xiaoyan@dicp.ac.cn (Y.X.); nnzhan@zjnu.edu.cn (N.Z.); lijie_zjnu@163.com (J.L.); 2 Dalian National Laboratory for Clean Energy, Dalian Institute of Chemical Physics, Chinese Academy of Sciences, 457 Zhongshan Road, Dalian 116023, China; 3 The State Key Laboratory of Catalysis, Dalian Institute of Chemical Physics, Chinese Academy of Sciences, 457 Zhongshan Road, Dalian 116023, China

**Keywords:** copper catalyst, hydrotalcite, stability, ethanol, butanol

## Abstract

The catalytic upgrading of ethanol into butanol through the Guerbet coupling reaction has received increasing attention recently due to the sufficient supply of bioethanol and the versatile applications of butanol. In this work, four different supported Cu catalysts, i.e., Cu/Al_2_O_3_, Cu/NiO, Cu/Ni_3_AlO_x_, and Cu/Ni_1_AlO_x_ (Ni^2+^/Al^3+^ molar ratios of 3 and 1), were applied to investigate the catalytic performances for ethanol conversion. From the results, Ni-containing catalysts exhibit better reactivity; Al-containing catalysts exhibit better stability; but in terms of ethanol conversion, butanol selectivity, and catalyst stability, a corporative effect between Ni–Al catalytic systems can be clearly observed. Combined characterizations such as XRD, TEM, XPS, H_2_-TPR, and CO_2_/NH_3_-TPD were applied to analyze the properties of different catalysts. Based on the results, Cu species provide the active sites for ethanol dehydrogenation/hydrogenation, and the support derived from Ni–Al–LDH supplies appropriate acid–base sites for the aldol condensation, contributing to the high butanol selectivity. In addition, catalysts with strong reducibility (i.e., Cu/NiO) may be easily deconstructed during catalysis, leading to fast deactivation of the catalysts in the Guerbet coupling process.

## 1. Introduction

Butanol is an important chemical feedstock that can be used as an organic solvent, chemical intermediate, and extractant in the cosmetics and pharmaceutical industries [1,2,3]. It is also one of the most promising alternatives to ethanol as a blending component of gasoline to improve fuel quality and reduce the emission of harmful gases [4,5,6]. Except for the well-established commercial technologies for the synthesis of butanol, including the oxo process (hydroformylation of propylene with syngas) [7] and the ABE process (fermentation of sugar-containing crops) [8], the catalytic upgrading of bioethanol to n-butanol is highly attractive due to the sufficient supply of bioethanol from renewable biomass [9,10,11,12].

**Scheme 1:**
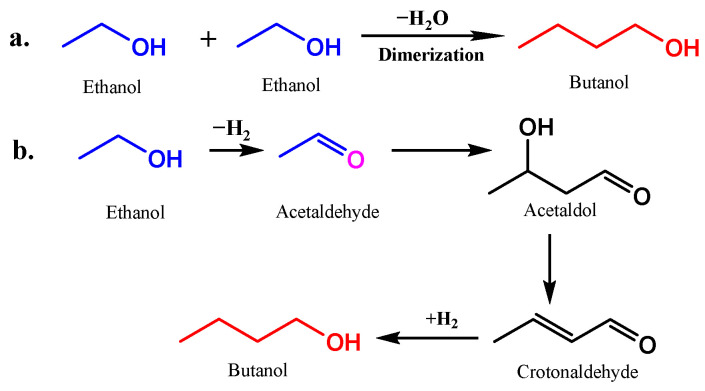
(**a**) The direct route and (**b**) Guerbet coupling process for ethanol transformation to butanol.

Generally, there are two accepted pathways for ethanol conversion to n-butanol, i.e., the direct coupling reaction of two ethanol molecules via dimerization to form n-butanol [13,14], and the Guerbet coupling process via multi-step tandem reactions with acetaldehyde and crotonaldehyde as intermediates (see Scheme 1) [15,16,17,18]. In the direct route, the β-H of the ethanol molecule is activated and reacted with another ethanol molecule through dehydration (Scheme 1a), which has been reported on solid-base catalysts or alkali cation zeolites [19,20]. But in the Guerbet coupling process, the C–C formation is accomplished by the aldol condensation route, consisting of dehydrogenation of ethanol, aldol condensation of acetaldehyde molecules, and subsequent hydrogenation of crotonaldehyde to form butanol (Scheme 1b) [14,17]. This reaction has been known for more than 100 years, and the dehydrogenation coupling of ethanol into n-butanol via C–C addition was first investigated by Guerbet over sodium alkoxide [21]. Inspired by the work, the coupling of ethanol into higher alcohols through the Guerbet coupling process via either homogeneous or heterogeneous catalysts has been reported by many researchers [22,23,24,25,26,27,28].

In homogeneous catalytic systems, high selectivity can be obtained by using iridium [29], ruthenium [30], and manganese [31] complexes as catalysts, accompanied by sodium ethoxide or strong bases. Whereas, although high conversions and selectivity have been obtained, great issues such as product purification, catalyst recovery, and waste treatment have become the main obstacles for industrial applications. The use of heterogeneous catalytic systems has been reported to be an alternative way to deal with the aforementioned problems [24,25,26,27,28]. But in heterogeneous systems, it is more challenging to synthesize butanol from ethanol because of the competitive side reactions such as ethanol dehydration to ethylene, ethanol dehydrogenative dimerization to ethyl acetate, and the Lebedev reaction to 1,3-butadiene [10,32].

Mixed metal oxides supported by transition metals are usually reported as efficient catalysts for the Guerbet coupling process [32,33,34,35,36,37,38,39,40], in which mixed metal oxides derive from layered double hydroxides (LDH). For example, MgAlO_x_ obtained by the thermal decomposition of Mg–Al–LDH is one of the most common supports for ethanol conversion to butanol [32,33,34]. A great number of Mg–Al–LDH-derived metal catalysts were designed and developed for this reaction [37,38,39]. For instance, G. W. Huber et al. investigated Mg–Al mixed oxide-supported Cu catalysts for the Guerbet coupling process [32]. They found that catalysts with low Cu loadings (0.1~0.6 wt.%) have high selectivity to C4+ alcohols (49~63%), whereas catalysts with high Cu loadings (>1.2 wt.%) are selective to esters and ketones. Zhang et al. prepared MgAlO_x_-supported Ru catalysts for ethanol conversion [33]. They discovered that the atomic Ru can promote ethanol dehydrogenation to acetaldehyde and the aldol condensation of acetaldehyde by adjusting the Ru dispersion and the acid–base properties [33]. Besides, Mg–Al mixed oxides supported by Pd [37], Ag [39], and Ni [40] catalysts are also synthesized and evaluated for ethanol coupling to butanol. However, the utilization of noble metal catalysts increases the final cost of the whole process. Thus, non-noble metal catalysts based on Ni and/or Cu are usually preferred.

Our group previously found that the non-noble metal Cu catalyst derived from Ni–Al–LDH (Cu/NiAlO_x_) exhibits good performance in ethanol conversion [36]. The Cu species with appropriate contents (>0.75 wt.%) was found to make a big difference in the catalytic performances. However, it is still ambiguous about the function of support for the reaction pathway. In fact, the support effect altered the properties of catalysts and impacted the catalytic performance during the conversion of ethanol [41,42]. Thus, in this work, to clarify the effect of support on ethanol conversion, four different supported copper catalysts (i.e., Cu/Al_2_O_3_, Cu/NiO, Cu/Ni_3_AlO_x_, and Cu/Ni_1_AlO_x_) were designed and prepared for the synthesis of n-butanol through the Guerbet coupling process. By adjusting the composition of the supports, the structure and properties of the catalysts were investigated in detail using X-ray powder diffraction (XRD), transmission electron microscopy (TEM), X-ray photoelectron spectroscopy (XPS), hydrogen temperature-programmed reduction (H_2_-TPR), and temperature-programmed desorption (TPD) of CO_2_/NH_3_. Based on these results, insights into the reasons for different catalytic performances and stabilities are revealed. We believe this work will provide good guidance for understanding the role of support and help to develop efficient catalysts with multiple functional sites for the Guerbet coupling process.

## 2. Results and Discussions

### 2.1. Catalytic Performances 

The catalytic performances of different copper catalysts (i.e., Cu/Al_2_O_3_, Cu/NiO, Cu/Ni_3_AlO_x_, and Cu/Ni_1_AlO_x_) were investigated in the upgrading of ethanol to butanol under the reaction conditions of T = 523 K, P_N2_ = 2 MPa, GHSV = 692 h^−1^ and LHSV = 4.8 h^−1^. The corresponding results are summarized in Table 1. From the results, the samples with high Ni content, i.e., Cu/Ni_3_AlO_x_ (Table 1, Entry 1) and Cu/NiO (Table 1, Entry 3), show high reactivity in the reaction, with an ethanol conversion of ~40%. While the samples with a high Al content, i.e., Cu/Ni_1_AlO_x_ (Table 1, Entry 2) and Cu/Al_2_O_3_ (Table 1, Entry 4), show lower reactivity with an ethanol conversion of ~30%. Notably, for the synthesis of butanol, Cu/Ni_3_AlO_x_ and Cu/Ni_1_AlO_x_ exhibit higher selectivity when compared with Cu/NiO and Cu/Al_2_O_3_, with values of 41.3% and 43.8%, respectively. In addition, higher alcohols such as hexanol were detected as the main byproduct, with selectivities of 8.7% and 11.4%, indicating the carbon chain growth via aldol condensation reaction is much easier on the Ni–Al catalytic systems. For the Cu/NiO, the ethanol conversion is relatively high (41.1%), but the butanol selectivity (37.0%) is not as high as that of the Cu/Ni_3_AlO_x_ and Cu/Ni_1_AlO_x_, indicating Al species have a positive effect on the synthesis of butanol. However, a pure Al_2_O_3_-supported Cu catalyst is not selective for obtaining butanol, with a selectivity of 24.1%, which agrees well with the previous literature since ethanol dehydration would happen over the Cu/Al_2_O_3_ catalyst to form ether and/or ethyl acetate due to the presence of a large number of acid sites [42,43].

### 2.2. Stability Tests 

**Figure 1:**
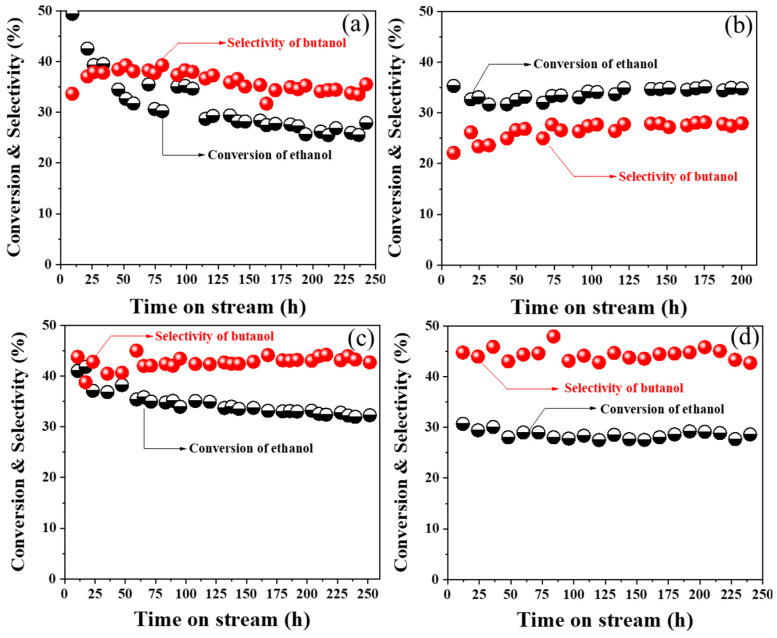
Stability tests of different supported Cu catalysts for ethanol conversion to butanol: (**a**) Cu/NiO; (**b**) Cu/Al_2_O_3_; (**c**) Cu/Ni_3_AlO_x_; (**d**) Cu/Ni_1_AlO_x_. Reaction conditions: catalyst: 2 g, temperature: 523 K, pressure of N_2_: 2 MPa, gas hourly space velocity (GHSV): 692 h^−1^, liquid hourly space velocity (LHSV): 4.8 h^−1^.

From an industrial point of view, the stability of catalysts is of great significance for ethanol applications. Thus, to measure the stability of the above-supported Cu catalysts, ethanol conversion and butanol selectivity were monitored continuously in the fixed-bed reactor for over 200 h. As seen in Figure 1, the samples with a high Ni content show obvious catalyst deactivation. Over the Cu/NiO (Figure 1a), the conversion of ethanol decreased from 49% to 26% after the reaction for 250 h time on stream (TOS), and the butanol selectivity decreased from 36% to 33%. On the contrary, over the Cu/Al_2_O_3_ (Figure 1b), the catalyst shows excellent stability. The transformation of ethanol was preserved throughout the reaction at about 34%, but the selectivity of butanol was maintained at a low level of 25~28%. For the Cu/Ni_3_AlO_x_ (Figure 1c), the stability of the catalyst has a certain improvement when compared with Cu/NiO, in which ethanol conversion decreased from 41% to 33% after 250 h TOS and the selectivity of butanol was preserved from 43~45%. Notably, for the Cu/Ni_1_AlO_x_ (Figure 1d), the conversion of ethanol was maintained at ~30%, and the selectivity of butanol stood at ~45%. As a consequence, in terms of ethanol conversion, butanol selectivity, and catalyst stability, copper supported on Ni_1_AlO_x_ shows the best performance. A corporative effect between Ni–Al catalytic systems can be clearly observed. Following characterizations and discussions will be supplied to make it clear the relationship between catalyst structure and catalytic performances.

### 2.3. Structural Properties of the Fresh and Spent Catalysts

**Figure 2:**
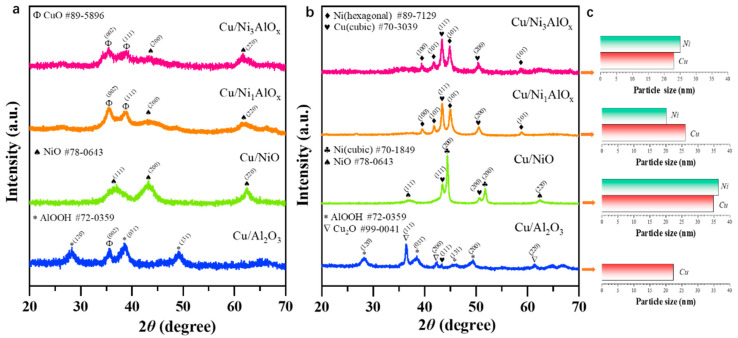
XRD spectra of different supported Cu catalysts (**a**) before and (**b**) after the catalytic tests; (**c**) average particle sizes of Ni and Cu over the spent catalysts.

X-ray diffraction (XRD) patterns show the crystal phases of different supported Cu catalysts before and after catalytic tests. As displayed in Figure 2a, the fresh Cu catalysts derived from Ni–Al hydrotalcite display the phase structure of both NiO (PDF#78-0643) and CuO (PDF#89-5896). After catalytic tests (Figure 2b), CuO and NiO over the spent Cu/Ni_3_AlO_x_ and Cu/Ni_1_AlO_x_ catalysts transformed into cubic Cu (PDF#70-3039) and hexagonal Ni (PDF#89-7129), implying the presence of a reducing atmosphere and the destruction of support during the ethanol coupling process. For the Cu/NiO, the NiO can be reduced to cubic Ni (PDF#70-1849), which is different from that of spent Cu/Ni_3_AlO_x_ and Cu/Ni_1_AlO_x_ (Figure 2b). The differences in the structure derive from the distinct arrangement of Ni atoms in the NiO and Ni–Al–LDH, which might change the support stability. Notably, for the Cu/Al_2_O_3_, the phase of AlOOH (PDF#72-0359) can be observed on both fresh and spent catalysts. Besides, Cu_2_O (PDF#99-0041) and cubic Cu (PDF#70-3039) can be observed over the spent Cu/Al_2_O_3_, indicating the support is relatively stable when compared with Ni-containing supports. By calculating the crystal sizes of Cu(111) and Ni(111) of spent catalysts via the Scherrer formula, the particle sizes of Cu and Ni are much smaller over the Al_2_O_3_ and/or Ni–Al–LDH-derived Cu catalysts than those of the Cu/NiO (Figure 2c), indicating the addition of Al species can prevent the agglomeration of metal particles, thus enhancing the stability of Cu species.

**Figure 3:**
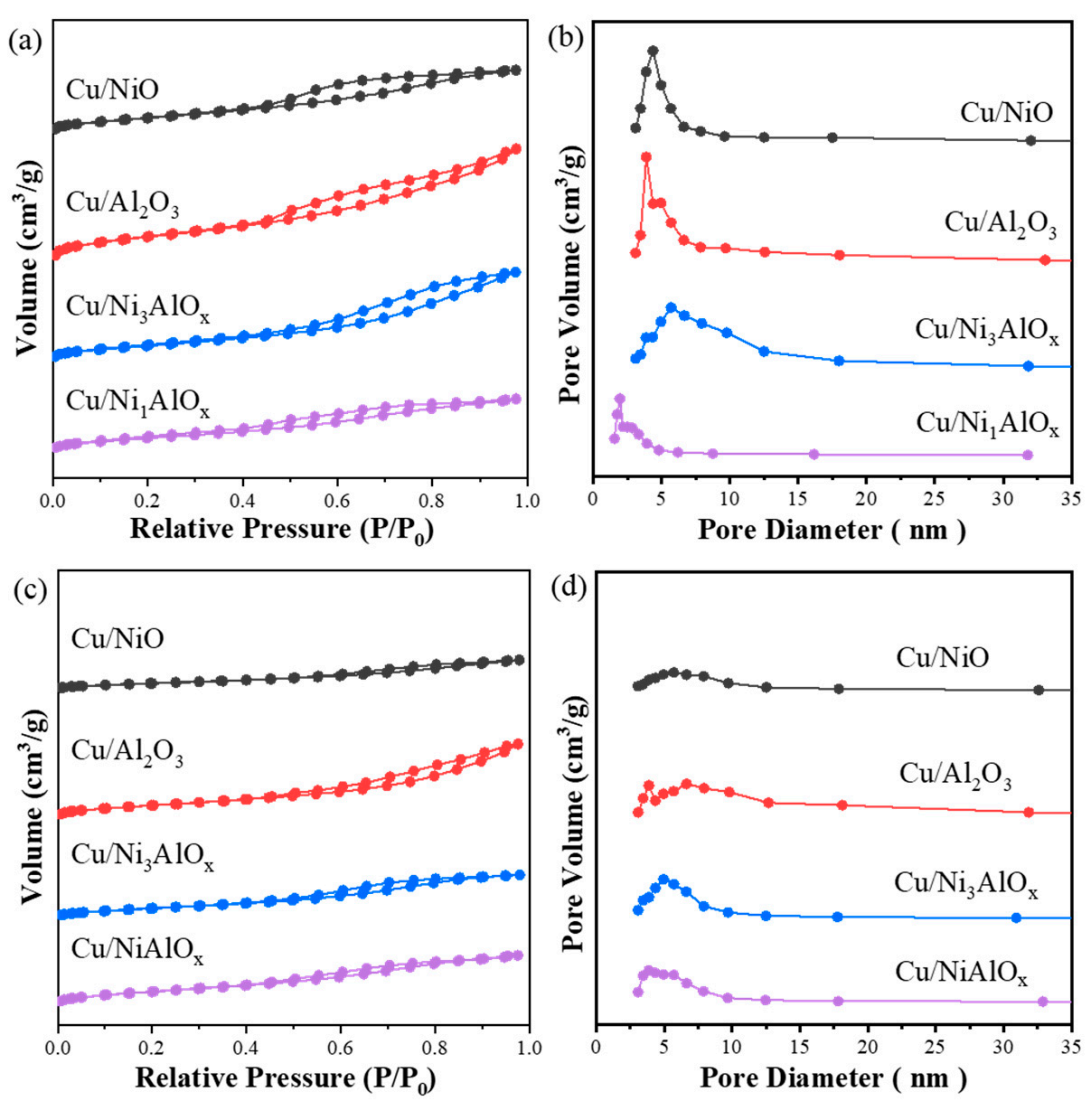
(**a**,**c**) N_2_ physical adsorption–desorption isotherms and (**b**,**d**) BJH pore size distributions of different supported Cu catalysts (**a**,**b**) before and (**c**,**d**) after catalytic tests.

N_2_ physical adsorption–desorption was applied to measure the pore structure of different supported Cu catalysts. The specific surface area (S_BET_), pore volume, and pore width are summarized in Table 2. From the data, the fresh Cu/Al_2_O_3_ catalyst has the largest S_BET_ of 351.6 m^2^/g and a pore volume of 0.48 cm^3^/g. Other catalysts show similar pore structures and S_BET_ in the range of 207.9~254.7 m^2^/g. The isothermal curves of the above catalysts are shown in Figure 3. From the curves, all materials displayed hysteresis loops of type H3 or H4 according to the IUPAC classification, indicating the presence of porous structures [44]. For the spent catalysts, the S_BET_ and pore volume of the supported Cu catalysts decreased a lot (Table 2 and Figure 3). Especially for the Cu/NiO, the S_BET_ sharply decreased from 228.7 m^2^/g to 79.4 m^2^/g, which is probably due to the formation of big metal particles blocking the channel of the supports. That agrees well with the XRD results. But for the Cu/Al_2_O_3_, Cu/Ni_3_AlO_x_, and Cu/Ni_1_AlO_x_, the reduction of S_BET_ is much smaller, indicating the catalysts are relatively stable. The contents of Cu, Ni, and Al were measured by inductively coupled plasma atomic emission spectroscopy (ICP-AES), from which all catalysts show similar Cu loadings of ~12 wt.% (Table 2), and the spent catalysts show no obvious metal leaching during catalysis.

**Figure 4:**
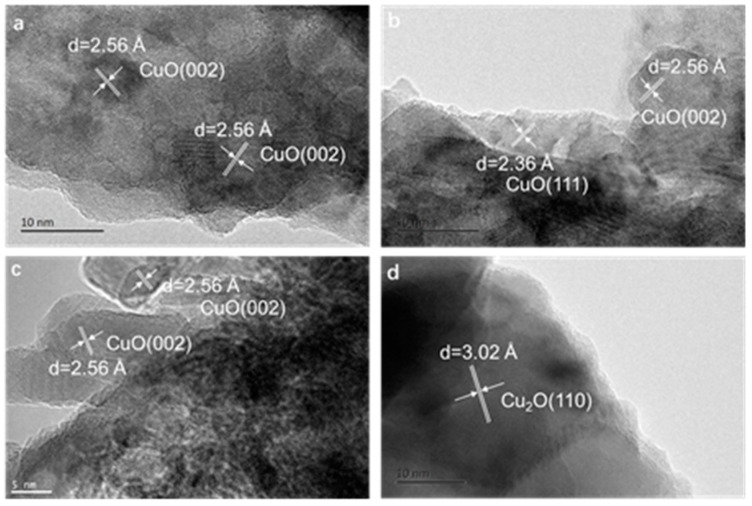
HRTEM images of different supported Cu catalysts: (**a**) Cu/Al_2_O_3_; (**b**) Cu/Ni_3_AlO_x_; (**c**) Cu/Ni_1_AlO_x_; and (**d**) spent Cu/Al_2_O_3_.

To analyze the microstructure of catalysts, high-resolution transmission electron microscopy (HRTEM) was employed on the above samples. As seen in Figure 4, a CuO(002) crystal phase with an interplanar spacing of 2.56 Å can be discerned in Cu/Al_2_O_3_ (Figure 4a), Cu/Ni_3_AlO_x_ (Figure 4b), and Cu/Ni_1_AlO_x_ (Figure 4c), which is in good line with XRD results (Figure 2a). However, the absence of an HRTEM image of Cu/NiO is due to the magnetism of the sample, which contaminated the electron microscope apparatus and did not show any efficient electronic images. In addition, the HRTEM image of Cu/Al_2_O_3_ after the reaction is presented in Figure 4d, in which Cu_2_O (110) crystal phases with an interplanar spacing of 3.02 Å were observed in most of the regions, which agrees well with the XRD results (Figure 2b). According to previous studies, Cu^0^ and Cu^+^ were generally regarded as the active sites for the Cu-catalyzed Guerbet coupling reaction [36,45]. Thus, Cu/Al_2_O_3_ was deduced to stabilize the active Cu species, displaying excellent stability in ethanol conversion reactions.

### 2.4. Insight into the Catalytic Performance and Stability

**Figure 5:**
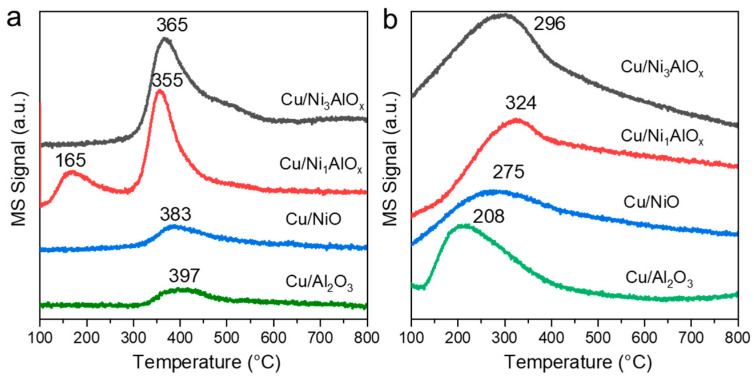
(**a**) CO_2_-TPD and (**b**) NH_3_-TPD profiles of different supported copper catalysts.

The acid–base property was considered to play a pivotal role in modulating the catalytic performance, especially in regulating the selectivity of the Guerbet coupling process [46,47,48]. Thus, to evaluate the acid–base property, CO_2_-TPD and NH_3_-TPD were conducted, respectively, for different supported Cu catalysts. As displayed in Figure 5a, all catalysts show a desorption peak of CO_2_ at 350~400 °C, implying the presence of medium-strong basic sites [49]. Notably, for the Cu/Ni_3_AlO_x_ and Cu/Ni_1_AlO_x_, the total amount of CO_2_ adsorption is obviously larger than that of the Cu/NiO and Cu/Al_2_O_3_, implying the catalysts possess much more basic sites than those of the Cu/NiO and Cu/Al_2_O_3_, which are derived from the abundant interlayer hydroxyl groups of Ni–Al–LDH. Based on the previous literature, the weak and medium basic sites are capable of catalyzing aldol condensation [33,35]. Thus, for Cu/Ni_3_AlO_x_ and Cu/Ni_1_AlO_x_, higher butanol selectivity was obtained when compared with Cu/NiO and Cu/Al_2_O_3_. 

In addition, except for the basic sites, the amounts of acid sites are also essential for regulating product distribution [32,50]. According to previous studies, too many acid sites are not favorable for obtaining high butanol selectivity owing to acid-catalyzed side reactions such as ethanol dehydration to ethyl acetate [42,43]. Herein, based on NH_3_-TPD measurements (Figure 5b), all catalysts possess a certain degree of acid sites, whereas Cu/Al_2_O_3_ exhibits the most amount of NH_3_ desorption, which is highly relevant to the phase of AlOOH [51]. According to our previous results (Table 1), Cu/Al_2_O_3_ shows the lowest butanol selectivity (24.1%). Meanwhile, a large amount of ethyl acetate (19.7%) was produced during catalysis. This phenomenon is attributed to the weak basicity and strong acidity of Cu/Al_2_O_3_ [42,43]. In comparison, Cu/NiO exhibits better performance with higher ethanol conversion (41.1%) and butanol selectivity (37%), compared to Cu/Al_2_O_3_. We deduced that the small amount of basic and acidic sites might form acid–base pairs to promote acetaldehyde condensation and generate more of the target product butanol [52]. Noteworthy, Cu/Ni_3_AlO_x_ and Cu/Ni_1_AlO_x_ catalysts derived from Ni–Al–LDH optimized the acid–base distributions and modified the balance of acid–base sites, favoring aldol condensation and obtaining the highest amount of butanol [36,46]. 

**Figure 6:**
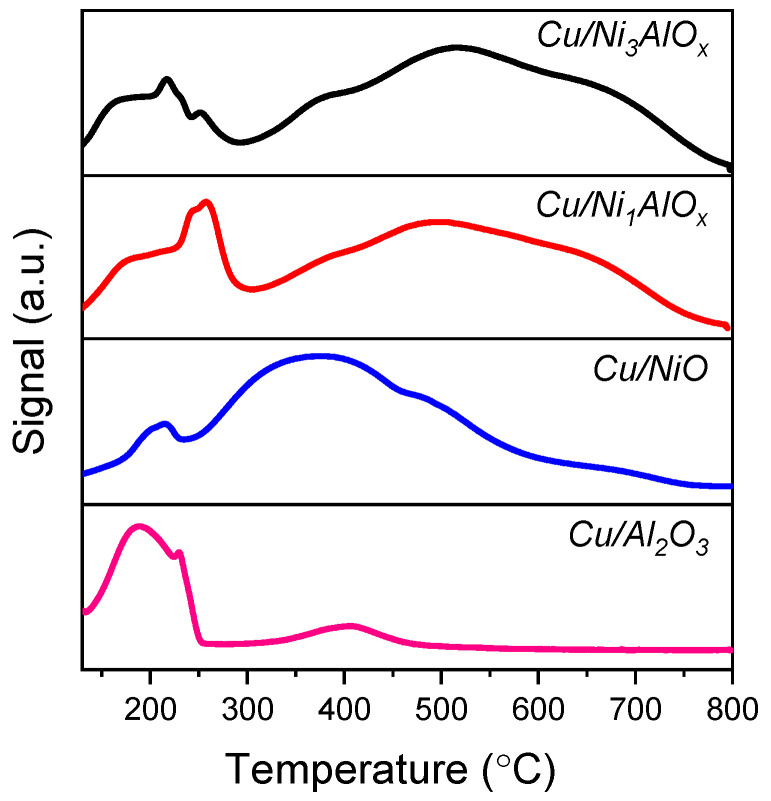
H_2_-TPR profiles of different supported Cu catalyst.

Except for acid–base sites, metal-active sites such as Cu and/or Ni were essential for the Guerbet coupling process, which involved the dehydrogenation of ethanol and the hydrogenation of crotonaldehyde [35,36]. From the previous results, part of CuO and NiO can be reduced to metallic Cu and metallic Ni (Figure 2), thus providing sufficient amounts of active sites for hydrogen transfer. In view of the catalytic performance and catalyst stability, Ni-containing samples afford better reactivity, and Al-containing samples afford better stability (Table 1 and Figure 1). Based on XRD (Figure 2) and HRTEM (Figure 4) results, Al-containing samples are more stable during catalysis when compared with Ni-containing samples because Al species can prevent the agglomeration of metal particles and benefit the stabilization of active Cu species (Figure 2b and Figure 4d). In considering the reducing atmosphere of ethanol conversion, the reducibility of the above catalysts was further evaluated by hydrogen temperature-programmed reduction (H_2_-TPR). As displayed in Figure 6, a wide and broad band in the range of 300~800 °C can be observed over the Ni-containing samples, which is assigned to the reduction peak of Ni^2+^ [36,40]. Whereas compared with Cu/Ni_3_AlO_x_ and Cu/Ni_1_AlO_x_, the reduction peak of Cu/NiO shifted to a lower temperature, indicating Cu/NiO is more easily reduced under the H_2_ atmosphere. Hence, the facile reducibility of Cu/NiO would generate more metallic Ni and lead to relatively poor stability during catalysis. For the Cu/Al_2_O_3_, no reduction peak of support can be observed, agreeing well with its excellent stability. Besides, an obvious consumption peak at 120~250 °C can be observed on all catalysts, which is assigned to the reduction peak of CuO [35,36]. Considering the reaction temperature is 250 °C, most of the Cu^2+^ species can be reduced to low valence, thus producing active Cu^0^/Cu^+^ species for the dehydrogenation and hydrogenation processes of the Guerbet coupling process [36,45].

**Figure 7:**
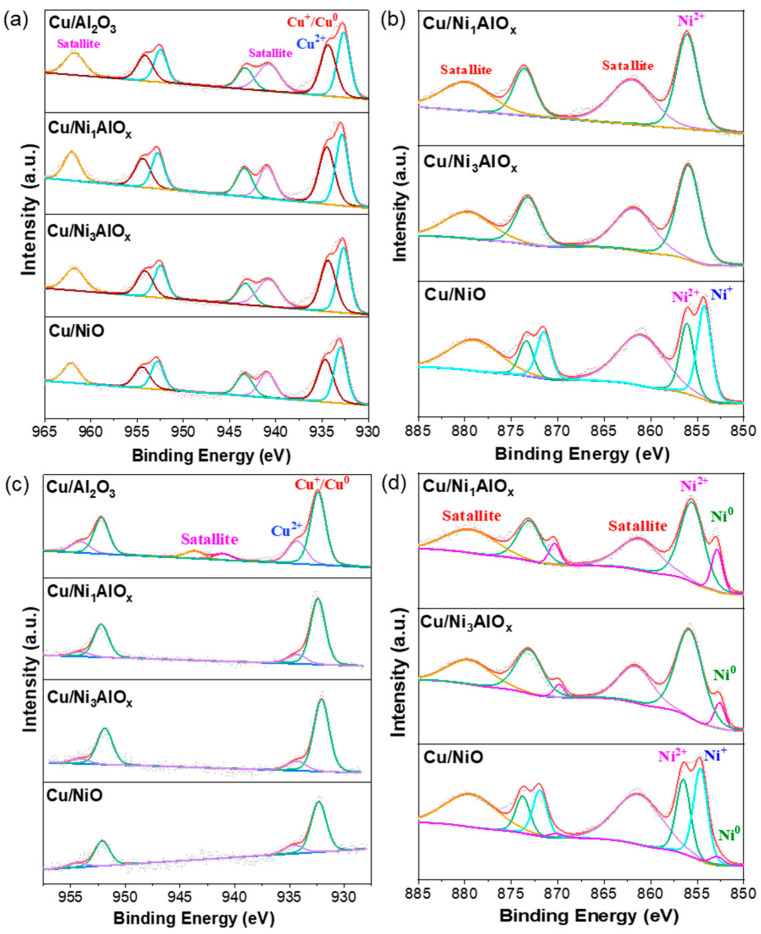
(**a**,**c**) Cu 2p and (**b**,**d**) Ni 2p XPS of different supported copper catalysts before (**a**,**b**) and after (**c**,**d**) catalytic tests.

The surface chemical states were further evaluated by X-ray photoelectron spectroscopy (XPS). The Cu 2p and Ni 2p XPS of the above catalysts before and after catalytic tests are shown in Figure 7a–d. From Cu 2p XPS (Figure 7a,c), most of the Cu^2+^ species over the spent catalysts (Figure 7c) were reduced to Cu^0^ and Cu^+^, as the peak positioned at 934.7 eV greatly decreased, accompanied by the disappearance or weakening of satellite peaks [35,36]. This result is also consistent with XRD, HRTEM, and H_2_-TPR results. To be noted, when compared with Ni-containing samples, Cu^2+^ over the Cu/Al_2_O_3_ is relatively difficult to reduce to Cu^+^/Cu^0^, indicating Cu species loaded on Al_2_O_3_ are more stable, which is in accordance with our previous results. In terms of Ni species (Figure 7b,d), the reduction of Ni^2+^ (855.6 eV) to Ni^0^ (852.69 eV) is clearly observed in all catalysts (Figure 7d), but for the Cu/NiO, a new peak positioned at 854.2 eV appeared over the fresh catalyst (Figure 7c), which is assigned to the presence of Ni^+^ [53]. Hence, the Cu/NiO has stronger reducibility compared to Cu/Ni_3_AlO_x_ and Cu/Ni_1_AlO_x_, which might lead to its poor stability during catalysis. This is also constant with H_2_-TPR results (Figure 6).

## 3. Materials and Methods

### 3.1. Materials 

Nickel nitrate hexahydrate (Ni(NO_3_)_2_·6H_2_O, ≥99.7%), copper nitrate trihydrate (Cu(NO_3_)_2_·3H_2_O, ≥99.7%), butanol (>99.5%), ethanol (>99.5%) and methanol (>99.5%) were purchased from Sinopharm. Sodium hydroxide (NaOH, 97 wt.%), sodium carbonate (Na_2_CO_3_, 99.5 wt.%), aluminum nitrate hydrate (Al(NO_3_)_3_·9H_2_O, 99 wt.%), ethyl acetate (98 wt.%), and ortho-xylene (>99.5%) were purchased from Aladdin Industrial Corporation. All chemicals are used directly without purification. Ultrapure water (18.2 MΩ) was used throughout this work.

### 3.2. Preparation of the Catalysts

NiAl-hydrotalcite (HT) with an Ni:Al molar ratio of 3:1 was prepared via the co-precipitation method. Typically, Ni(NO_3_)_2_·6H_2_O (0.28 mol) and Al(NO_3_)_3_·9H_2_O (0.09 mol or 0.28 mol) were mixed by adding 400 mL of water to obtain solution A. Na_2_CO_3_ (0.347 mol) and NaOH (1.320 mol) were dissolved in 400 mL of water to obtain solution B. In a water bath at 70 °C, solution A was dropwise added to solution B via a constant flow pump with a flow rate of 50 mL/min. Then, the gel was continuously stirred at 70 °C and aged for 24 h. After being cooled to room temperature, the precipitate was filtered, washed, dried at 80 °C for 8 h, and then ground to 100 mesh to obtain Ni_3_Al-HT or Ni_1_Al-HT.

The supports containing only Ni or Al were prepared with a similar process. Firstly, Ni(NO_3_)_2_·6H_2_O (0.28 mol) or Al(NO_3_)_3_·9H_2_O (0.28 mol) were mixed with 400 mL of water to obtain solution A. Then, the solution was dropwise added to solution B (400 mL), which contained 0.347 mol of Na_2_CO_3_ and 1.320 mol of NaOH in a water bath at 70 °C. Afterwards, the gel was continuously stirred at 70 °C for 24 h and cooled down to room temperature. The precipitate was filtered, washed with deionized water, dried at 80 °C for 8 h, and then ground to 100 mesh to obtain the supports of Ni(OH)_2_ or Al(OH)_3_. 

Supported Cu catalysts were prepared via the hydrothermal deposition precipitation method. At first, 5.00 g of the above supports was added to 100 mL of deionized water. The pH value of the solution was adjusted to 10 by adding 0.16 M of Na_2_CO_3_. Then, 2.2 g of Cu(NO_3_)_2_·3H_2_O was dissolved in 20 mL of water, and the pH value of the precipitating solution was also maintained at 10. The above solutions were then intensively mixed by stirring and heated to 80 °C in a water bath, followed by aging for 2 h. The solid catalyst was obtained after filtering, washing with deionized water, drying overnight at 80 °C, and further calcining in air at 300 °C for 2 h before the reaction test with a heating rate of 5 °C/min. The catalysts, which are denoted as Cu/NiO, Cu/Al_2_O_3_, Cu/Ni_3_AlO_x_, and Cu/Ni_1_AlO_x_, have theoretical Cu loadings of 12 wt.%.

### 3.3. Catalytic Evaluation

The catalytic conversion of ethanol into butanol was performed in a micro-reaction system on a stainless steel fixed-bed reactor (inner diameter, 10.0 mm; length, 660 mm), equipped with a thermocouple and a mass flow controller for the control of reaction temperature and gas flow rate. Typically, specific amounts of catalysts were placed in the middle of the reactor, and then the pressure of the system was increased and maintained with a back-pressure regulator. After purging with N_2_ for 30 min, the temperature was programmed to increase to the specific value with a heating rate of 5 °C/min. Then, ethanol was introduced by using a plunger pump (NP-KX-210) with a constant liquid hourly space velocity (LHSV). The reaction products were analyzed offline with an Agilent 7890B chromatograph equipped with an HP-5 capillary column (30 m × 0.32 mm) and a flame ionization detector (FID). O-xylene was used as an internal standard for the quantification of the liquid products. Products were identified with a GC–MS from Agilent (Agilent 7890B-5977B MSD) with the same HP-5 column. 

For the catalytic upgrading of ethanol, the conversion was calculated as moles of ethanol reacted to the moles of ethanol fed to the reactor. The selectivity of each product was calculated as the ratio of moles of carbon in the target product to the moles of carbon in the ethanol that reacted. These calculations can be given by the following formulas:

$$\mathrm{Conversion}\;(\%)\;=\;\frac{\mathrm{Moles}\;\mathrm{of}\;\mathrm{ethanol}\;\mathrm{reacted}}{\mathrm{Moles}\;\mathrm{of}\;\mathrm{ethanol}\;\mathrm{fed}\;\mathrm{to}\;\mathrm{the}\;\mathrm{reactor}}\;\times \;100\;$$

$$\mathrm{Selectivity}\;(\%)\;=\;\frac{\mathrm{Moles}\;\mathrm{of}\;\mathrm{carbon}\;\mathrm{in}\;\mathrm{the}\;\mathrm{target}\;\mathrm{product}}{\mathrm{Moles}\;\mathrm{of}\;\mathrm{carbon}\;\mathrm{in}\;\mathrm{ethanol}\;\mathrm{reacted}}\;\times \;100$$

### 3.4. Characterization

The actual contents of different elements were determined by inductively coupled plasma atomic emission spectroscopy (ICP-AES) on the IRIS Intrepid II XSP instrument (Thermo Electron Corporation, Waltham, MA, USA). N_2_ physisorption isotherms were measured at 77 K on a Quantachrome NT3LX-2 instrument after degassing the samples at 300 °C for 2 h. The specific surface area, total pore volumes, and pore size distributions were calculated according to the BET and BJH methods. X-ray diffraction (XRD) analysis was carried out on a PW3040/60 X’Pert PRO (PANalytical, Almelo, The Netherlands) diffractometer in a scanning angle (2θ) range of 10–90°. High-resolution transmission electron microscopy (HRTEM) was recorded on a JEM-2100F microscope (Tokyo, Japan) at 200 kV. The samples were prepared by dispersing the catalyst powder in ethanol via ultrasonication onto a micromolybdenum TEM grid. X-ray photoelectron spectroscopy (XPS) was conducted on a Thermo ESCLALAB 250Xi spectrometer equipped with a monochromatic Al and double anode Al/Mg target as a radiation source. The binding energies were calibrated by using the C 1s peak (284.8 eV) as the reference. Hydrogen temperature-programmed reduction (H_2_-TPR) experiments were performed on a Micromeritics Autochem II 2920 equipped with a thermal conductivity detector (TCD) and mass spectrometry (MS). Before the measurement, 100 mg of the samples was treated at 300 °C for 60 min in a quartz U-tube reactor under a He gas flow of 30 mL/min. After cooling to room temperature, a 10% H_2_/Ar flow (30 mL/min) was introduced, and the sample was heated to 800 °C at a ramping rate of 10 °C/min. Temperature-programmed desorption of CO_2_ and NH_3_ (CO_2_/NH_3_-TPD) measurements were also conducted on a Micromeritics Autochem II 2920. Prior to the tests, about 100 mg of the catalyst was outgassed at 300 °C for 1 h in a U-type quartz tube reactor with a He gas flow of 50 mL/min. Next, the samples were cooled down to 100 °C, and then pulses of CO_2_ or NH_3_ were introduced into the tube to ensure saturation of the samples. The temperature-programmed desorption was performed up to 800 °C with a heating ramp of 10 °C/min. The signal of the desorbed CO_2_ or NH_3_ was recorded by the MS simultaneously.

## 4. Conclusions

In conclusion, four different copper catalysts, i.e., Cu/Al_2_O_3_, Cu/NiO, Cu/Ni_3_AlO_x_, and Cu/Ni_1_AlO_x_, were prepared and investigated in the continuous catalytic transformation of ethanol into butanol. From the results, a corporative effect between Ni–Al catalytic systems can be clearly observed in terms of ethanol conversion, butanol selectivity, and catalyst stability. The results from XRD and HRTEM indicated that the addition of Al might stabilize the active Cu species and prevent the agglomeration of metal particles, leading to its excellent stability in ethanol conversion. H_2_-TPR and XPS results implied that Ni-containing samples have strong reducibility, which might destroy the structure and lead to the deactivation of the catalyst. In addition, an appropriate balance of acid–base sites was shown to make a large difference in product selectivity, favoring catalyzing aldol condensation and obtaining the product butanol, which can be proved by CO_2_/NH_3_-TPD results. Based on these findings, catalysts with multiple functional sites for the Guerbet coupling process need to be designed. We believe this work will provide good guidance for understanding the role of Ni and Al in supporting and helping to develop efficient catalysts for the ethanol conversion reaction.

## Figures and Tables

**Table 1:** Catalytic activities of different supported Cu catalysts for ethanol conversion.

Entry	Catalysts	Conversion (%) ^a^	Selectivity (%) ^a^						
			Butanol	Ethyl Acetate	Ether	Butaldehyde	Ethyl Butyrate	Butyl Acetate	Hexanol
1	Cu/Ni_3_AlO_x_	39.0	41.3	4.3	2.9	4.7	4.2	12.0	8.7
2	Cu/Ni_1_AlO_x_	30.0	43.8	7.7	3.4	3.8	2.5	5.0	11.4
3	Cu/NiO	41.1	37.0	3.1	6.4	4.4	1.6	10.2	1.0
4	Cu/Al_2_O_3_	32.9	24.1	19.7	2.7	4.4	4.6	3.1	3.3

Reaction conditions: catalyst: 2 g, temperature: 523 K, pressure of N_2_: 2 MPa, gas hourly space velocity (GHSV): 692 h^−1^, liquid hourly space velocity (LHSV): 4.8 h^−1^. ^a^ Conversion and selectivity were calculated by the results of average values within 48 h detected by gas chromatography.

**Table 2:** Textural properties of supported Cu catalysts.

Entry	Catalysts	Cu (wt.%) ^a^	Ni (wt.%) ^a^	Al (wt.%) ^a^	Ni/Al Molar Ratio ^a^	S_BET_ (m^2^·g^−1^) ^b^	Volume (cm^3^·g^−1^) ^b^	D_pore_ (nm) ^b^
1	Cu/NiO	12.6	64.8	-	-	228.7	0.28	4.35
2	Cu/Al_2_O_3_	12.8	-	38.8	-	351.6	0.48	3.86
3	Cu/Ni_3_AlO_x_	12.3	48.4	7.2	3.1	254.7	0.39	5.69
4	Cu/Ni_1_AlO_x_	12.9	32.9	13.5	1.1	207.9	0.21	1.93
5	Cu/NiO ^c^	11.6	71.3	-	-	79.4	0.13	4.94
6	Cu/Al_2_O_3_ ^c^	11.3	-	36.0	-	168.0	0.32	3.86
7	Cu/Ni_3_AlO_x_ ^c^	12.0	49.4	7.5	3.0	125.5	0.19	4.96
8	Cu/Ni_1_AlO_x_ ^c^	12.3	39.6	16.6	1.1	162.4	0.20	3.86

^a^: The actual loadings of Cu, Ni and Al were evaluated by ICP-AES measurement; ^b^: specific surface area (S_BET_), total pore volume, and average pore size were calculated from N_2_ physisorption isotherms; ^c^: spent catalysts.

## Data Availability

Data are available upon request from the corresponding authors.
